# Investigating Possible Trans/Intergenerational Associations With Obesity in Young Adults Using an Exposome Approach

**DOI:** 10.3389/fgene.2019.00314

**Published:** 2019-04-05

**Authors:** Jean Golding, Steven Gregory, Kate Northstone, Yasmin Iles-Caven, Genette Ellis, Marcus Pembrey

**Affiliations:** Population Health Sciences, Bristol Medical School, University of Bristol, Bristol, United Kingdom

**Keywords:** ALSPAC, smoking, obesity, intergenerational, transgenerational, exposome, fat mass

## Abstract

Animal experiments demonstrate ways in which an exposure in one generation can be reflected in a variety of outcomes in later generations. In parallel human observational studies have shown associations between grandparental and parental exposures to cigarette smoking and/or nutrition and growth and survival of the grandchild. These studies have controlled for just a few confounders selected *ad hoc*. Here we use an exposome approach (using all available measures of exposure) to determine trans/inter-generational factors that may be important in studying environmental factors associated with fat mass in young human adults. The study takes advantage of the rich data available in the Avon Longitudinal Study of Parents and Children (ALSPAC). We test associations with features of grandparents (G0) and the childhood of the parents (G1) of 24-year olds (G2). We hypothesized that intergenerational associations would be revealed, particularly with exposure to cigarette smoke, and that these would vary with the sexes of all three generations. The study exposome analyzed 172 exposures to the maternal line and 182 to the paternal line. A series of stepwise regression analyses reduced the initial 40 unadjusted factors (*P* < 0.05) to eight independent features on the maternal line, and of 26 on the paternal line to five. We found strong associations between the father starting to smoke cigarettes regularly before age 11 and increased fat mass in his adult children (unadjusted = +7.82 [95% CI +2.75, +12.90] Kg; adjusted = +11.22 [+5.23, +17.22] Kg); this association was stronger in male offspring. In addition, when the paternal grandmother had smoked in pregnancy her adult granddaughters, but not grandsons had elevated mean fat mass (interaction with sex after adjustment, *P* = 0.001). The exposome technique identified other factors that were independently associated with fat mass in young adults. These may be useful in identifying appropriate confounders in other more proximal analyses, but also may identify features that may be on epigenetic pathways leading to increased fat mass in subsequent generations. We acknowledge that the results need to be replicated in other cohorts and encourage further linkage of outcomes with previous generational exposures, particularly along the paternal line.

## Introduction

It is universally recognized that overweight/obesity is a public health problem, particularly because it is on the causal pathway to type 2 diabetes and the metabolic syndrome, with associated co-morbidities and mortality (Bray and Bellanger, 2006). In the United States in 2000 it was estimated that about 47 million adults had the metabolic syndrome, almost a quarter of the adult population (Ford et al., 2002) and there is evidence indicating that the prevalence has been increasing over time (Mozumdar and Liguori, 2011). The increasing epidemic of overweight/obesity is not confined to western populations – it was estimated that, worldwide, there were as many as 1.1 billion individuals affected in the early 2000s (James et al., 2004). An improved understanding of the pathways by which individuals become obese is clearly an important preliminary to identifying possible preventative strategies. It is now clear that transmission of information to the next generation is not simply genetic inheritance of DNA and its variations, together with so-called “cultural inheritance” acquired through learning. There is transmission of molecular information via the gametes about earlier parental (and possibly grandparental) experiences and exposures both external and internal. Based on animal experiments, a prime candidate mechanism is some form of epigenetic inheritance, including the paternal transmission of non-coding RNAs in sperm (Hur et al., 2017). However, molecular analyses are not the focus of the present study.

Most population studies of obesity have used the individual’s body mass index as the outcome measure. However, by definition, this is dependent on body weight, and consequently includes the weight of bone and muscle as well as fat. In this study we have therefore concentrated on the amount of fat stored within the body (the fat mass) which is the component of body mass that results in ill health.

The concept of identifying details of environmental exposures to the parents and grandparents in order to assess effects on the study children was developed in the design of the ALSPAC birth cohort (Golding et al., 2001). Although the possibility of an exposome analysis was intended, this technique was formally developed by Wild (2005). The ALSPAC study proposed using many measures of the external environment, including social and psychological as well as chemical and demographic exposures. In contrast, Wild emphasized the use of internal measures of exposure using biomarkers. However, he pointed out that “at its most complete, the exposome encompasses life-course environmental exposures (including lifestyle factors), from the prenatal period onward. Developing reliable measurement tools for such a complete exposure history is extremely challenging. Unlike the genome, the exposome is a highly variable and dynamic entity that evolves throughout the lifetime of the individual.”

**FIGURE 1:**
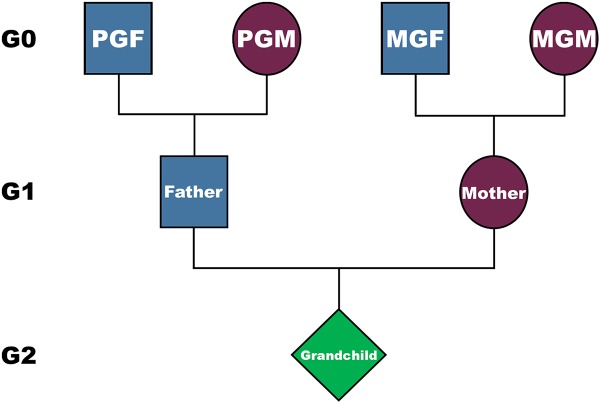
The structure of the inheritance, and possible genetic and non-genetic pathways from grandparents (G0) to their grandchildren (G2), via their parents (G1). PGM is the paternal grandmother, PGF the paternal grandfather, MGM the maternal grandmother, and MGF the maternal grandfather.

There has been growing recognition of the importance of the exposome in elucidating possible causal factors as well as identifying potential confounders (Buck Louis et al., 2013; Louis et al., 2017), and many have emphasized the importance of starting an exposome early in pregnancy (Vrijheid et al., 2014; Robinson and Vrijheid, 2015). Here we investigate the importance of starting the exposome even earlier, by assessing features of the grandparents and the parents long before the conception of the offspring of interest. Building on our earlier studies we hypothesize that intergenerational/ transgenerational environments experienced by grandparents (G0) or parents (G1) in their own childhoods may be associated with G2 adult outcomes such as obesity. We use data from the Avon Longitudinal Study of Parents and Children (ALSPAC) and concentrate on features of the lives of the grandparents prior to the birth of the parents, and on the childhoods of each parent. Based on our previous studies of fat mass in this cohort (at ages 9–17), which controlled for features proximal to the birth of the study child (parity of the mother at birth, parental education, maternal smoking during pregnancy, housing tenure, and paternal smoking at conception) (Golding et al., 2014; Northstone et al., 2014), we hypothesized that the smoking habit of the paternal grandmother (G0) in the pregnancy that resulted in the birth of the study father (G1), and the prepubertal age at his starting to smoke regularly will both be identified as independently associated with the fat mass of the study child (G2) in adulthood. We were hypothesis free in regard to other exposures. We did, however, hypothesize that any smoking-related associations identified would vary according to the sex of the grandparent, the parent and the study offspring (see Figure 1).

## Materials and Methods

### Population Sample

As indicated, the data employed is from the ALSPAC, a study that enrolled women pregnant in a defined area (Avon, England), who had an expected date of delivery between April 1991 and December 1992. The aims of the study were to determine the environmental and genetic factors that influence the health and development of the offspring (Golding et al., 2001). During pregnancy, the parents were each asked to complete detailed questions concerning their own parents and their childhood. The initial number of pregnancies enrolled was 14,541. Of these initial pregnancies, there was a total of 14,676 fetuses, resulting in 14,062 live births and 13,988 children who were alive at 1 year of age. There was a range of social circumstances with 40% of the partners working in manual occupations, 73% living in owner occupied homes; median mother’s age was 28 with their partners approximately 2 years older. Only 5% of the study offspring had a non-white background (similar to the Avon population at the time), and 45% were first-born.

The parents and their surviving offspring have been followed up ever since (Boyd et al., 2013; Fraser et al., 2013). Data continued to be collected at various time-points using self-completion questionnaires, assays of biological samples, hands-on measurements, and linkage to other data sets, and is available to researchers upon application to the ALSPAC Executive. The study website contains details of all the data that are available through a fully searchable data dictionary and variable search tool: http://www.bristol.ac.uk/alspac/researchers/our-data/.

### The Outcome Measure

At age 24, the ALSPAC study offspring (G2) were invited to a clinic for a number of tests including a DXA scan. In all, 9997 individuals were invited and 4026 (40.3%) attended. After excluding from measurement those who were pregnant; whose weight was greater than 159 Kg; those who had undergone a recent nuclear medicine investigation with persistent radioactivity or a recent radiological investigation using contrast media, 3607 individuals had a valid measure of total fat mass for analysis.

A Lunar Prodigy narrow fan beam densitometer was used to perform a whole body DXA scan where bone, lean and fat masses are measured. The procedure was clearly explained to the participant and written consent was obtained before proceeding. The participant was asked to lie on the Prodigy couch (in light clothing without any metal fastenings). The arm of the machine moved over the participant and two sources of X-ray scanned him/her. The participant was reassured throughout the scan and encouraged to keep as still as possible. The data from the DXA scan were used to calculate the fat mass.

### The Environmental Measures

Information on the grandparents (G0) and the childhood of each parent (G1) was collected on structured questionnaires mailed to the study parents during pregnancy. They therefore completed them in their own homes, and were able to contact relevant family members if they did not know particular details. In total there were 172 variables considered that may have affected the maternal line, and 182 for the paternal line; the difference in numbers was due to the inadvertent omission of some of the variables in the questionnaires to the study mothers (G1). The various measures used in the analyses are described in the Supplementary Material. Briefly, for this exposome we have formed the following groups of exposures: For the maternal line of inheritance: (i) the study mothers’ parents – the maternal grandparents (G0); (ii) the childhood of the study mother (G1). For the male line: (i) the study fathers’ parents – the paternal grandparents (G0); (ii) the childhood of the study father (G1). The exposures to the G1 generation in childhood concerned many aspects of the environment, including the composition of the home, stability of their parents (G0), maternal care given (G0), traumatic events including various types of abuse, attitudes to school, deaths or serious illness or accidents to family members, as well as recollections of happiness/unhappiness. For both generations, the years of birth as well as the ages at which events occurred were given. See Supplement C for a full description of the data considered. For these analyses the only feature of the G2 generation that was considered was their sex and their 24-year-old fat mass (as the outcome).

### The Statistical Approach

We performed an “exposome” analysis. This is similar to the approach taken in genome-wide association (GWAS) studies, being hypothesis free. Rather than testing already formulated hypotheses, a GWAS examines associations with as many as two million genetic markers for predetermined statistical significance levels and then attempts to replicate the findings in other studies. In general, no adjustment is made for confounders.

**FIGURE 2:**
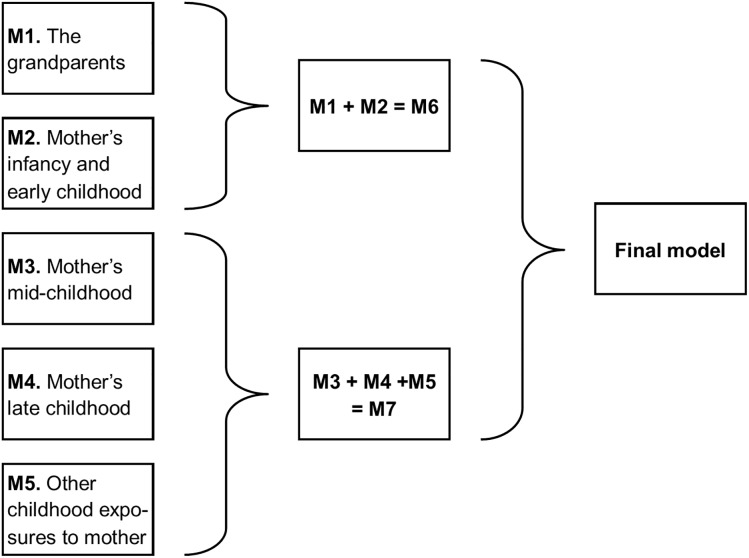
Structure of the analysis of the exposures to the maternal line.

**FIGURE 3:**
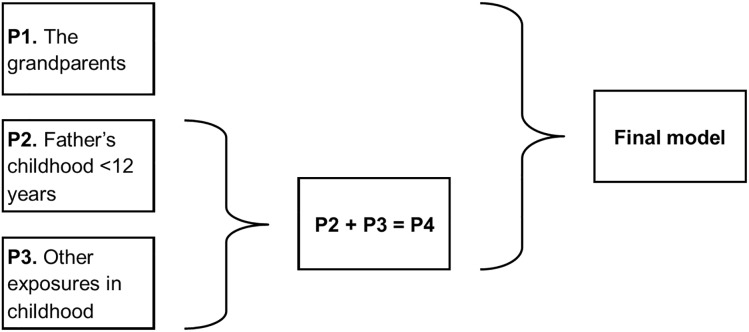
Structure of the analysis of the exposure to the paternal line.

In the present study, which is a hypothesis-free generator in regard to the environmental factors, we first assessed the relationship between fat mass and each exposure variable. In order to ensure that we did not suffer from type II errors, we did not correct for multiple testing at this stage, but included all variables associated with the outcome at *P* < 0.05. For the smoking variables, where we had a prior hypothesis, we used *P* < 0.10. In order to look for patterns, the environmental variables were divided and considered in logical groupings. For the parental exposures these were largely dictated by the ages at which they were experienced. For each set of analyses of the different subgroups we used backward stepwise linear regression. We then used a logical set of sequences, first identifying the independent variables from each subgroup and combining the variables in a number of steps into a final model. Importantly the analyses were carried out separately for exposures along the maternal and paternal lines (see Figure 2, 3).

## Results

Of the 3607 grandchildren for whom there were DXA measurements, total fat mass ranged from 5.31 to 64.86 Kg, with mean of 22.07 Kg and standard deviation of 8.84 Kg. Below we consider the two different inheritance pathways separately: the maternal line comprising the maternal grandparents, and the mother’s own exposure in childhood, and the paternal line consisting of the paternal grandparents and the father’s childhood exposures.

### The Maternal Line

#### Unadjusted Associations and Subgroup Analyses

The first step in the exposome of the maternal line was to assess the degree to which the exposures were significantly associated with mean fat mass. In all, 40 (24%) variables were significantly associated, a third of them (8% of all variables) at the *P* < 0.0001 level – far exceeding expectation. The 40 variables are considered in 6 subgroups (Table 1 and Supplement A).

**Table 1:** Summary of the numbers of variables considered, and numbers of these showing unadjusted significant associations with the 24-year-old’s fat mass.

Subgroups considered	No. tested	No. *P* < 0.05–0.01	No. *P* < 0.01–0.001	No. *P* < 0.001–0.0001	No. *P* < 0.0001	Total (%) *P* < 0.05
**Maternal line**						
MGPs	14	0	0	2	7	9 (64)
M infancy	12	0	1	0	3	4 (33)
M early chdhd	28	5	0	0	0	5 (18)
M mid-chdhd	34	3	2	0	2	7 (21)
M late chdhd	31	7	1	0	1	9 (29)
M other chdhd	53	3	2	1	0	6 (11)
All maternal line	172	18	6	3	13	40 (24)
**Paternal line**						
PGPs	14	4	2	0	1	7 (50)
F infancy	11	0	1	0	0	1 (9)
F early chdhd	31	0	0	0	0	0
F mid-chdhd	35	5	2	0	0	7 (20)
F late chdhd	32	1	2	0	0	3 (9)
F other chdhd	59	3	4	0	1	8 (2)
All paternal line	182	13	11	0	2	26 (14)


*F, father (G1); M, mother (G1); MGP, maternal grandparents (G0); chdhd, childhood; PGP, paternal grandparents (G0).*

##### M1: The maternal grandparents before the birth of the mother

Of the 14 variables considered, nine (64%) were associated with fat mass at a *P*-value of <0.001. These comprised: the year of birth of each of the maternal grandparents, their levels of education, their social classes based on their occupations, their ages when the mother was born, and whether the maternal grandfather (but not the grandmother) had been a smoker (Supplementary Analysis AM1). Stepwise analyses revealed that just two variables had clearly accounted for all the other variables – these comprised the maternal grandmother’s levels of education (grandchildren of grandmothers with lower academic levels having grandchildren who had, on average, 1.99 Kg more fat), and the maternal grandfathers’ years of birth (the more recently he had been born, the higher the fat mass of +0.11 Kg per year of birth).

##### M2: The mother in infancy and early childhood (<6 years)

As noted in Table 1, of the 12 infancy variables 4 (33%) were associated with the fat mass in the next generation – the mother’s year of birth, whether or not she was born in Avon, whether born post-term and whether she had been breast fed. In contrast only five (18%) of the early childhood variables were significantly associated (*P* < 0.05): these comprised three types of accidents (badly scalded, head injury, nearly drowned) as well as a summary variable of severe injury (which included these and other accidents/injuries), and whether her parents had divorced or separated. These variables were combined with the infancy variables and offered to a step-wise regression; all the four infancy variables remained, as did two of the five early childhood variables (badly scalded and nearly drowned). With the exception of breast feeding all exposure variables were associated with increased fat mass in generation G2 (Supplementary Analysis AM2).

##### M3: The mother in mid-childhood (6–11 years)

There were 34 variables considered for exposures in mid-childhood and seven (21%) were significantly associated with fat mass – four were related to persons within the study mother’s household at this time (her mother, father, stepfather, and grandfather), one related to an accidental injury (fractured arm), and two to biological experiences (a history of wetting during the day, and onset of menarche prior to age 12). Stepwise regression using these seven variables indicated that only one dropped out (father in the household). All the adjusted variables showed a significant increase in fat mass with the exception of the presence of the mother’s mother in the home at this time point; her grandchildren had accumulated 2.58 Kg less fat than expected on average by the age of 24 (Supplementary Analysis AM3).

##### M4: The mother in late childhood (>11 years)

Late childhood was defined as ages 12–15. There were 31 exposure variables covering this time period, and nine were statistically significant when unadjusted. Of these, two concerned the residents in the home at the time, one an accident (head injury), one concerned starting to smoke regularly, and five concerned events such as being admitted to hospital, her parent having an accident, becoming pregnant, being suspended from school and her parents separating. Only two of these survived the stepwise analysis – beginning smoking and her parent having an accident. Both were associated with increased fat mass in the next generation (Supplementary Analysis AM4).

##### M5: Other childhood measures

The 53 exposures considered in this subgroup have no specific age group attached. Only six (11%) were statistically associated prior to adjustment: two were concerned with schooling [whether attended a special school, and the total number of schools attended (<3 vs 3+)], three were related to non-parental care (in care, living in a children’s home and living with grandparents), and the mother’s assessment of her mother as being over-protective. Upon stepwise regression, just three variables remained: attending a special school, attending <3 schools altogether, and living with her grandparents. Attending <3 schools was associated with a lower level of fat mass (Supplementary Analysis AM5).

#### Combining the Results From the Subgroup Analyses

The analytic structure for the combination of the results from each subgroup analysis is shown diagrammatically in Figure 2 and described below.

##### M6: Combining the results from the subgroup analyses M1 and M2

This analysis considered the two variables that were identified in the maternal grandparent analyses, together with the four from infancy and two from the early childhood analyses; five of these eight variables remained in the analysis: both the grandparent variables, the post-term variable from the infancy variables, and both the injuries from early childhood (Supplementary Analysis AM6).

##### M7: Combining the analyses from subgroups M3, M4, and M5

The six variables concerning the study mother from mid-childhood, two from late childhood and three non-specified age were offered together to a step-wise analysis (Figure 2). Of the 11 variables, seven survived the combined subgroup analysis – maternal grandmother, and grandfather in the household in mid-childhood; fractured arm mid-childhood; menarche <12; began smoking <16, her parent had a major accident when she was aged 12–15; she attended <3 different schools in total (Supplementary Analysis AM7).

##### M8: The final model for the maternal line

The final model combines the 12 variables surviving analyses M6 and M7. Of these eight survived the final stepwise regression and are depicted in Table 2. It can be seen that the two variables concerning the maternal grandparents dominate in terms of significance levels – the less well-educated the maternal grandmother the greater the fat mass (+1.98 Kg) accumulated by the grandchild on average, and the more recently the grandfather was born the greater the level of fat (+0.1 Kg per increasing year of birth). Ignoring the two variables with *P*-values >0.01, the other possible factors associated with the outcome are whether the mother (G1) was badly scalded in early childhood, whether her grandfather was in the household in mid-childhood, whether she commenced menarche before age 12 and whether she was smoking regularly by age 15.

**Table 2:** The final adjusted models for the maternal line: mean difference in fat mass (95% CI) Kg: all grandchildren.

Variable considered	Grandparents and early childhood	Later childhood	Final model
MGM’s education <O- level	1.93 (1.08, 2.78)****		1.98 (1.11, 2.86)****
MGF’s year of birth^a^	0.11 (0.06, 0.16)****		0.10 (0.05, 0.15)****
M born post-term	2.79 (0.72, 4.87)**		2.27 (0.07, 4.47)*
M badly scalded <6 years	4.56 (0.68, 8.43)*		5.42 (1.40, 9.43)**
M nearly drowned <6 years	5.25 (0.52, 9.98)*		-
MGM in M’s household 6–11		-2.18 (-4.13, -0.23)*	-
Grandfather in M’s household 6–11		3.04 (0.76, 5.31)**	4.46 (1.78, 7.14)***
M fractured arm 6–11		2.22 (0.43, 4.00)*	-
M’s periods started <12 years		2.09 (1.07, 3.12)****	1.74 (0.59, 2.88)**
M began smoking 12–15		2.27 (0.99, 3.56)***	2.11 (0.61, 3.61)**
M’s parent had a major accident 12–15		2.18 (0.21, 4.16)*	2.79 (0.57, 5.01)*
M attended <3 different schools <17		-1.00 (-1.80, -0.20)*	-
N (*R*^2^) in model	2277 (2.52%)	2792 (2.11%)	2100 (3.96%)


*^*a*^Treated as continuous; ^∗^*P* < 0.05; ^∗∗^*P* < 0.01; ^∗∗∗^*P* < 0.001; ^∗∗∗∗^*P* < 0.0001. M, mother; MGM, maternal grandmother; MGF, maternal grandfather.*

##### Possible sex interactions

The study had as its aims to determine whether any of the findings were associated with different associations between the sexes of the grandchildren. We therefore examined the associations between each of the exposures in the final model and fat mass to determine whether there was evidence of a difference that warranted a more formal analysis. Table 3 shows that of the 12 variables that were considered in Table 2, there were three that showed signs of a sex interaction among the unadjusted data: the MGF’s year of birth, which showed a much stronger trend for the granddaughters compared with the grandsons, whether the mother’s grandmother had been living with her 6 to 11-year-old daughter (reduction in fat mass among the granddaughters), and her grandfather living with the study mother when she was 6–11 (increase in fat mass among the granddaughters) (Table 3).

**Table 3:** Difference (in Kg) between the sexes in unadjusted associations of items in the three models of the maternal line in Table 2.

Variable considered	Male G2s b (95% CI)	Female G2s b (95% CI)
MGM’s education <O-level	1.41 (0.29, 2.51)^∗^	1.75 (0.70, 2.80)^∗∗^
MGF’s year of birth^a^	0.78 (0.19, 1.36)^∗∗^	1.22 (0.72, 1.71)^∗∗∗∗^
M born post-term	2.48 (-0.15, +5.10)-	0.54 (-0.53, 1.62)-
M badly scalded <6 years	5.56 (1.20, 9.91)^∗^	3.34 (-1.04, 7.72)-
M nearly drowned <6 years	3.04 (-2.95, 9.03)-	4.81 (0.02, 9.61)^∗^
MGM in M’s household 6–11	0.17 (-2.43, 2.77)-	-3.75 (-5.89, -1.60)^∗∗∗^
Grandfather in M’s household 6–11	0.43 (-2.64, 3.50)	3.60 (1.01, 6.20)^∗∗^
M fractured arm 6–11	3.12 (0.59, 5.65)^∗^	2.16 (0.02, 4.30)^∗^
M’s periods started <12 years	2.10 (0.74, 3.47)^∗∗^	2.45 (1.21, 3.70)^∗∗∗^
M began smoking 12–15	1.72 (-0.06, 3.50) (^∗^)	2.51 (1.07, 3.95)^∗∗∗^
M’s parent had a major accident 12-15	2.57 (0.05, 5.09)^∗^	2.17 (-0.02, 4.55) (^∗^)


*^*a*^Treated as continuous; -*P* ≥ 0.10; (^∗^)*P* < 0.10; ^∗^*P* < 0.05; ^∗∗^*P* < 0.01; ^∗∗∗^*P* < 0.001; ^∗∗∗∗^*P* < 0.0001. M, mother; MGM, maternal grandmother; MGF, maternal grandfather.*

### The Paternal Line

#### Unadjusted Associations and Subgroup Analyses

As demonstrated in Table 1, there were fewer significant factors associated with the paternal line compared with the maternal line (26 vs 40, respectively), and many fewer with an adjusted *P*-value <0.001 (2 vs 16). Although the structure of the analysis was planned to mirror that used for the maternal line, the numbers of variables in infancy (one) and early childhood (0) precluded complex analyses of these data sets, and the one infancy variable was included with the mid-childhood model.

##### P1: The paternal grandparents before the birth of the father

Similar data were found to be associated with the fat mass of their grandchildren as was found for the maternal line, with positive trends with year of birth, and education levels of both grandparents; in addition there were associations with the paternal grandmother’s age at the birth of the study father, whether the paternal grandfather had ever smoked and his social class, both the latter showed positive associations with grandchild’s fat mass. Stepwise regression indicated that just two variables dominated – the year of birth of the PGF and his social class.

A prior hypothesis had been that there would be an association between the paternal grandmother smoking in pregnancy and grandchild’s fat mass, and that this would be sex dependent. We therefore added this variable with an interaction term to the result of the above analysis and showed that there was a borderline interaction (*P* = 0.089), in the presence of which the association with the PGF’s year of birth showed a *P*-value of >0.10 (Supplementary Analysis AP1x). This variable was therefore omitted, and the analysis rerun. This indicated a more significant interaction between PGM smoking in pregnancy and the sex of the grandchild (*P* = 0.005) (Supplementary Analysis AP1xx). The association with social class remained strong.

##### P2: The study father from birth to age 11 inclusive

Unlike the associations found in the maternal line, here there was only one significant association with aspects of infancy (being born in Avon), and no associations with aspects of early childhood. We therefore combined the Avon data with those relating to the father at ages 6–11 (Supplementary Analysis AP2). Of the nine variables involved, five survived the stepwise regression analyses – each showed increased fat mass if the study father had a history of being born in Avon; having a history of nearly drowning or having a head injury when aged 6–11; whether the father truanted from school before age 11; and whether the father had started smoking regularly prior to age 11.

##### P3: The study father and other childhood measures

There were 11 variables included in this subgroup (Supplementary Analysis AP3), but only four remained in the model – these indicated increased fat mass in study children whose father had a step-father in the home when he was aged 12–15; whether the study father was frequently absent from school after the age of 11; whether he had spent time in a Children’s Home; and whether the father considered that his own mother was generally unstable. Each of these variables was associated with increased fat mass in their adult offspring.

#### Combining the Results From the Subgroup Analyses (Figure 3)

##### P4: The father’s childhood: combining results from P2 and P3

There were nine features of childhood that survived the different subgroup analyses. These were offered together to a stepwise regression analysis (Supplementary Analysis AP4), but only one variable dropped out (presence of step-father in the home at age 12–15.

##### P5: Final model: combining results from P1xx with P4

The addition of the variables from P1 to P4 resulted in the two in P1 remaining (i.e., PGF’s social class and the interaction between PGM smoking in pregnancy and sex of the grandchild. From the paternal features, five variables dropped out of the analysis: the father being born in Avon; whether he was nearly drowned; whether he often truanted before the age of 11; whether he had spent time in a children’s home and the stability of his mother. Consequently, there were just five variables left in the Final Model comprising whether the father had a head injury in mid-childhood; whether often absent from school aged 11+; the PGF’s social class; whether the father had started smoking before age 11; and an interaction between the grandmother smoking in pregnancy and sex of her grandchild (Table 4 and Supplementary Table AP5).

**Table 4:** The final adjusted models for the paternal line: mean difference in fat mass (95% CI) Kg.

Variable considered	Grandparents only	Father in childhood	Final model
PGF’s social class (per class)	0.82 (0.44, 1.19)****		0.64 (0.28, 1.01)***
PGM smoked prenatally: interaction with sex^a^	1.67 (-0.26, 3.60) (*)		3.21 (1.34, 5.08)***
F born in Avon		1.17 (0.23, 2.11)*	-
F nearly drowned aged 6–11		4.06 (0.52, 7.60)*	-
F had head injury aged 6–11		2.32 (0.83, 3.80)**	2.06 (0.63, 3.49)***
F started smoking aged <11		10.17 (3.72, 16.61)**	11.22 (5.23, 17.22)****
F often truanted from school <11		2.68 (0.82, 4.54)*	-
F often absent from school 11+		2.68 (0.82, 4.54)**	2.80 (1.08, 4.52)***
F spent time in Children’s home		6.96 (0.16, 13.76)*	-
Degree of instability in F’s mother		1.27 (0.32, 2.21)**	-
No. (*R*^2^) in model	2628 (5.25%)a	1902 (3.27%)	1975 (6.33%)a


*^*a*^Note that sex of grandchild is included in these models; (^∗^) *P* < 0.10; ^∗^*P* < 0.05; ^∗∗^*P* < 0.01; ^∗∗∗^*P* < 0.001; ^∗∗∗∗^*P* < 0.0001. F, father; PGM, paternal grandmother; PGF, paternal grandfather.*

##### Possibility of other sex interactions

In Table 5 we examine the unadjusted associations of all variables in the final model with mean fat mass for each sex of the grandchild to determine the possibility of sex interactions, so that these can be tested. Only one is obvious – the paternal grandmother smoking in pregnancy (which has already been shown to result in a significant interaction *P* < 0.001 on adjustment). Although the relationship with the study father starting to smoke regularly before the age of 11 showed a mean difference of 8 Kg between male and female offspring, numbers were small and the difference was not significant.

**Table 5:** Difference (in Kg) between the sexes in unadjusted associations of items in the three models of the paternal line in Table 4.

Variable considered	Male G2s b	(95% CI)	Female G2s b	(95% CI)	Interaction present
PGM smoked prenatally	-0.47 (-1.63, 0.70)		1.51 (0.49, 2.53)**		Yes^∗^
F born in Avon	1.60 (0.20, 2.61)**		1.52 (0.57, 2.49)**		
F nearly drowned aged 6–11	4.65 (-0.09, 9.39) (*)		2.49 (-1.73, 6.71)		
F had head injury aged 6–11	1.64 (-0.62, 3.89)		-0.06 (-3.42, 3.29)		
F started smoking aged <11	13.22 (4.83, 21.61)**		5.01 (-1.19, 11.20)		
F often truanted from school <11	3.73 (-2.23, 9.70)		4.52 (0.39, 8.65)*		
F often absent from school 11+	2.09 (0.06, 4.11)*		2.73 (1.02, 4.45)**		
F spent time in Children’s home	9.06 (2.14, 15.98)*		5.80 (-1.18, 12.77)-		


*(^∗^)*P* < 0.10; ^∗^*P* < 0.05; ^∗∗^*P* < 0.01; –*P* > 0.10. F, father; PGM, paternal grandmother.*

## Discussion

The aim of this study was to determine whether there were features of the environment occurring prior to the conception of the offspring (G2) that were independently associated with the accumulation of their body fat at 24 years after the physiological upheavals of puberty Although this was largely a hypothesis free approach, we did hypothesize that two features of the paternal line would be identified. These prior hypotheses stemmed from both observations of G2’s development into adolescence (Golding et al., 2014; Northstone et al., 2014) and independent studies of inter/trans-generational responses in humans (Pembrey et al., 2014). Indeed, we identified strong associations with both (i) a history of the grandmother smoking in the pregnancy resulting in the father, which showed a predicted interaction with the sex of the grandchild (with female but not male grandchildren having increased fat mass), and (ii) a history of the study father starting to smoke regularly before the onset of puberty (i.e., <11 years) and this indicated that his sons were at greater risk of increased fat mass than his daughters.

The remaining analyses were hypothesis free. The maternal line was dominated by associations between the MGF’s year of birth (the more recent, the greater the increase in fat, particularly among the granddaughters), and the MGM’s educational attainment; neither of these two associations were changed by allowing for features of the study mother during childhood. In contrast, for the paternal line, there was no association with the grandparents’ years of birth but there was a strong association with the PGF’s social class as measured using his occupation categorization and indicating that the ‘lower’ the occupation status the greater the grandchild’s adult fat mass (Supplement B). It is unclear whether these features of the grandparents indicate poorer social and intellectual status through the generations, or whether more specific (and unmeasured) influences are at play.

In regard to the parents’ childhood, we suggest that the stage of the final model is an appropriate time to confine attention to findings that have a *P*-value <0.01 as the factors with greater *P*-values are more likely to have arisen by chance. For the maternal line this assumption identified just four variables from the mothers’ childhoods: if she had a history of being badly scalded in early childhood, whether a grandfather was resident in the mother’s household when she was in mid-childhood, if she had begun smoking regularly in childhood, and whether her onset of menarche was <12 years. It is feasible that two of these had a psychological component that might affect the next generation in that bad scalds may result in visible scars with consequent lower self-confidence (Rumsey and Harcourt, 2007), and a grandfather in the home may be demanding of attention and the mother (G1) may have been deprived of appropriate care. The other two factors may suggest a biological pathway to obesity in their offspring, since women who have had an early menarche are at increased risk themselves of putting on weight (e.g., Lee et al., 2007), and women who have started smoking in childhood are likely to still be smoking when they reproduce – a known risk factor for obesity in their offspring (Behl et al., 2012).

The final model for the paternal line was dominated by five factors: the two related to smoking and the PGF’s social class have already been discussed above; only two other factors concerned the father’s childhood: having had a head injury in mid-childhood, and a history of often being absent from school in later childhood (adolescence). Whether these are on a causal pathway or have occurred by chance must await results from other multigenerational datasets.

### The Statistical Approach

Although this is primarily a hypothesis free approach, unlike the GWAS method (which mainly combines all factors into one analysis and uses a method to determine at what *P*-value to determine significance), we have taken a more structured view by considering data along a time-related path starting with grandparents, and then the different ages of parental childhood exposures. The motive for considering that age at exposure may be relevant to any form of non-genetic inheritance derives from the Överkalix studies (Bygren et al., 2001; Pembrey et al., 2006). The authors indicated that exposures of the paternal grandfathers in what the authors referred to as “the slow growth period” (approximately equal to the ages 6–11 years used in this study) appeared to have an influence on outcomes in future generations. Support for this exposure sensitive period comes from a recent publication using the larger Uppsala Multigeneration Study (Vågerö et al., 2018).

We purposefully chose not to use a correction for multiple testing such as a Bonferroni or False Discovery Rate correction due to the exploratory nature of the analyses; we did not want to omit potentially important exposures because of type 2 errors. In the event, on the maternal line there were 40 out of 172 factors identified initially with *P* < 0.05; with the subsequent structured set of analyses this was reduced to just eight independent factors (Table 2). For the paternal line, the initial 26 of 182 variables significant at *P* < 0.05 were reduced to five (Table 4).

In general, replication in comparable multigenerational cohorts is necessary to verify associations revealed by exposome analyses. Establishing and following up such multigenerational cohorts is in itself a huge challenge, let alone exploring potential cross-generational causal pathways to obesity. As mentioned in the Introduction, the prime candidate mechanism for transmitting exposure information to the next generation(s) is some form of epigenetic inheritance via the gametes. Relevant to obesity is the convincing evidence from rodent experiments that small non-coding RNAs, particularly tRNA fragments (tRFs), in sperm can result in inter/trans-generational inheritance in relation to both dietary protein restriction (Sharma et al., 2016) and high fat diet (Chen et al., 2016). Interestingly, using their obese prediabetic mouse model system, Cropley et al. (2016) were able to demonstrate the transmission down the male line of the *propensity* to become obese. The FI sons of obese founder males exhibited defects in glucose and lipid metabolism, but only upon a post-weaning dietary challenge. F1 males transmitted these defects/propensity to their own male progeny (F2) in the absence of the dietary challenge.

The above experiment was not designed to look at sex differences, but many have noted sex-specificity with respect to both the transmitting parent/ancestor and the outcome in offspring (reviewed in Pembrey et al., 2014). Such sex-specificity was hypothesized in the current exposome study and possible sex interactions were noted in the maternal line. In the paternal line analysis, the sex interactions were just the two hypothesized from earlier observations of the development of the G2 offspring. The link between paternal grandmother smoking in pregnancy with granddaughters having increased fat mass, and fathers smoking before puberty having sons with higher fat mass, is compatible with X and Y chromosome transmission. However, the field has a long way to go before explaining the sex-specific observations in mechanistic detail, especially in humans.

An important feature of smoking as a candidate exposure is that tobacco causes DNA damage (Laubenthal et al., 2012; Beal et al., 2017). The DNA damage response system results in the DNA in the nucleus being less tethered/restrained, with this increased mobility facilitating access to DNA repair complexes (Mekhail, 2018). This “mobility” in turn may compromise the control of DNA repeat sequences and transposable elements by DNA methylation and repressive chromatin states in the germline and in turn in the emerging nervous system of the early embryo of the next generation. Thus, in considering the cross-generational causal pathways to obesity and altered metabolism, one must consider both direct effects of tobacco exposure and also the DNA damage response system. The latter may result in wider effects in the next generation. In this context intergenerational responses to high dose nicotine in male mice has shown an increased tolerance of, not just nicotine, but other xenobiotics in male (but not female) offspring, suggesting a generic response (Vallaster et al., 2017).

### Strengths and Weaknesses

The strengths of this study are as follows: (a) it is population based and not restricted to any particular group of individuals; (b) the environmental information collected was prior to any knowledge concerning the weight gain of the study subjects; (c) although information on the grandparents and parental childhoods was collected retrospectively, this was obtained through structured paper questionnaires completed by the study parents in their own home, with encouragement to ask their own parents when appropriate.

There are some disadvantages to the study: (i) Although this is a population study, as with all longitudinal cohorts over time biases are introduced due to attrition; (ii) Apart from the two variables for which we had a prior hypothesis, we only tested for sex differences in the variables that were statistically significant in the final models. However, it is equally possible that there are exposures for which there are significant interactions but which do not have a significant main effect, and will therefore have been missed.

## Conclusion

We have used the data on factors occurring long before the conception of the study participant (to parents in childhood and to grandparents) to assess the possible influences on the level of fat mass in the child/grandchild as a young adult. Using an exposome allows for determining a time-related pathway. The methodology revealed associations at the age of 24 years that are in line with our previous published findings concerning fat mass in adolescence; namely associations of increased fat mass with both a history of the paternal grandmother smoking prenatally and of the father starting to smoke regularly before age 11. We suggest that future intergenerational studies would benefit from using exposome techniques to identify unexpected associations which may provide both clues to possible mechanisms, and/or determine appropriate confounders to be taken into account.

## Ethics Statement

This study was carried out in accordance with the recommendations of ALSPAC Ethics and Law Committee and the Local Research Ethics Committees. The protocol was approved by the ALSPAC Ethics and Law Committee.

## Author Contributions

JG had the idea. JG and MP wrote the first draft of the manuscript. SG and GE analyzed the data. All authors contributed to the final version of the manuscript.

## Conflict of Interest Statement

The authors declare that the research was conducted in the absence of any commercial or financial relationships that could be construed as a potential conflict of interest.
