# Supplementary material for: Investigating Possible Trans/Intergenerational Associations With Obesity in Young Adults Using an Exposome Approach
Source: Front Genet. 2019 Apr 5;10:314. doi: 10.3389/fgene.2019.00314 (PMC6459952; doi:10.3389/fgene.2019.00314)
Supplement: Supplementary file 1 [file Data_Sheet_1.PDF]

Supplement A. Step-by-step analyses (backward stepwise) of the factors initially associated with fat mass at age 24, and the results of the backwards step-wise regression. Each table shows the unadjusted associations and the results of stepwise regression, giving the mean fat mass difference in grams as the regression coefficient (b) with 95% confidence intervals (CI).

The Maternal Line

Supplementary Analysis AM1: The maternal grandparents prior to the birth of the mother

	Unadjusted			Adjusted		
**Variable**	**N**	**b [95% CI]**	**P**	**N**	**b [95% CI]**	**P**
MGM's year of birth (c772yr)	3243	129.2 [83.9, 174.4]	**2.3×10^{-8}**			
MGM's education <O-Level (c686a)	2721	1815 [1026, 2604]	**6.8×10^{-6}**	2459	1990 [1178, 2802]	**1.6×10^{-6}**
MGM's age at mother's birth (c772)	3243	-112.8 [-176.9, -48.8]	**0.001**			
MGM's social class (c_sc_mgm)	1954	643.1 [304.0, 982.3]	**2.1×10^{-4}**			
MGF's year of birth (c783yr)	76	119.6 [77.1, 162.0]	**3.6×10^{-8}**	2459	111.4 [65.5, 157.3]	**2.0×10^{-6}**
MGF's education <O-Level (c706a)	2577	1654 [840.8, 2468]	**6.9×10^{-5}**			
MGF ever smoked (b682)	3348	1355 [547.5, 2162]	**0.001**			
MGF's age at mother's birth (c783)	3076	-114.6 [-170.9, -58.3]	**6.8×10^{-5}**			
MGF's social class (c_sc_mgf)	2904	559.4 [253.6, 865.3]	**3.4×10^{-4}**			
						R^2=1.78%

Supplementary Analysis AM2: Mother in infancy and early childhood (<6 years)

	Unadjusted			Adjusted		
**Variable**	**N**	**b [95% CI]**	**P**	**N**	**b [95% CI]**	**P**
Mother's year of birth (mzyear)	3573	225.1 [148.6, 301.6]	**8.7 x 10^{-9}**	2570	192.9 [98.7, 287.0]	**6.0 x 10^{-5}**
Mother was born in Avon (d245)	3177	1730 [998.8, 2462]	**3.7 x 10^{-6}**	2570	1287 [453.0, 2121]	**0.003**
Mother born post-term (dw034)	3362	2700 [904.4, 4496]	**0.003**	2570	2649 [665.0, 4633]	**0.009**
Mother was breast fed (c624)	3009	-1446 [-2229, -662.4]	**3.0 x 10^{-4}**	2570	-1351 [-2192, -509.8]	**0.002**
Mother was badly scalded aged <6 (d085_0)	3380	4029 [826.7, 7232]	**0.014**	2570	4561 [1033, 8089]	**0.011**
Mother had a head injury aged <6 (d121_0)	3179	2889 [234.4, 5543]	**0.033**			
Mother nearly drowned aged <6 (d097_0)	3391	4363 [467.7, 8258]	**0.028**	2570	7366 [2660, 12071]	**0.002**
Mother suffered any injury aged <6 (d_dv1)	3443	1477 [342.7, 2611]	**0.011**			
Mothers parents divorced aged <6 (d388_0)	3449	2366 [360.9, 4371]	**0.021**			
						R^2=2.81%

Supplementary Analysis AM3: Mother in mid-childhood (6-11 years)

	Unadjusted			Adjusted		
**Variable**	**N**	**b [95% CI]**	**P**	**N**	**b [95% CI]**	**P**
MGM in household when aged 6-11 (d421)	3449	-2563 [-4257, -868.8]	**0.003**	2996	-2582 [-4445, -719.2]	**0.007**
MGF in household when aged 6-11 (d424)	3449	-2249 [-3451, -1047]	**2.5 x 10^{-4}**			
Stepfather in household when aged 6-11 (d436)	3449	2329 [87.0, 4571]	**0.042**	2996	2535 [222.6, 4848]	**0.032**
Grandfather in household when aged 6-11 (d454)	3449	2588 [556.2, 4619]	**0.013**	2996	3084 [856.3, 5311]	**0.007**
Mother wetting during day mid-childhood (d274b)	3449	4455 [627.7, 8283]	**0.023**	2996	5728 [1611, 9845]	**0.006**
Mother fractured arm when aged 6-11 (d091_6)	3309	2685 [1015, 4355]	**0.002**	2996	2676 [952.3, 4400]	**0.002**
Mother started having periods aged 6-11 (d010_6)	3121	2227 [1261, 3193]	**6.4 x 10^{-6}**	2996	2123 [1139, 3108]	**2.4 x 10^{-5}**
						R^2=1.77%

Supplementary Analysis AM4. Mother in late childhood (12-15 years)

Variable	N	Unadjusted b [95% CI]	P	N	Adjusted b [95% CI]	P
MGM in household when aged 12-15 (d422)	3449	-2656 [-4345, -967.4]	**0.002**			
MGF in household when aged 12-15 (d425)	3449	1249 [230.8, 2267]	**0.016**			
Mother had a head injury aged 12-15 (d121_12)	3179	3032 [398.3, 5666]	**0.024**			
Mother began smoking when aged 12-15 (b651_12)	3483	2518 [1373, 3663]	**1.7×10^{-5}**	3397	2537 [1369, 3705]	**2.1×10^{-5}**
Mother admitted to hospital aged 12-15 (c407a)	3462	756.2 [12.4, 1500]	**0.046**			
Mother's parent had accident aged 12-15 (c410a)	3462	2175 [385.4, 3964]	**0.017**	3397	2224 [228.8, 4019]	**0.015**
Mother became pregnant when aged 12-15 (c414a)	3462	2160 [417.3, 3903]	**0.015**			
Mother's parents separated aged 12-15 (c417a)	3462	1282 [239.2, 2324]	**0.016**			
Mother suspended from school aged 12-15 (c427a)	3462	2781 [277.1, 5285]	**0.030**			
						R^2=0.71%

Supplementary Analysis AM5. Mother in other childhood situations

Variable	N	Unadjusted b [95% CI]	P	N	Adjusted b [95% CI]	P
Mother attended <3 schools (c440)	3344	-894.0 [-1626, -162.5]	**0.017**	3206	-873.8 [-1621, -126.7]	**0.022**
Mother attended a special school (d273)	3401	4358 [1032, 7684]	**0.010**	3206	4330 [836.1, 7823]	**0.015**
Mother was in care (d385)	3293	4846 [1190, 8501]	**0.009**			
Mother lived with her grandparents (d392)	3429	3066 [1190, 4943]	**0.001**	3206	2896 [923.4, 4869]	**0.004**
Mother stayed in a children's home (d402a)	3404	4814 [934.0, 8694]	**0.015**			
Mother's mother was over-protective (d728)	3449	98.5 [11.4, 185.6]	**0.027**			
						R^2=0.63%

Supplementary Analysis AM6. The maternal grandparents and mother's early childhood (tables AM1 and AM2 combined)

		**Unadjusted**			**Adjusted**	
**Variable**	**N**	**b [95% CI]**	**P**	**N**	**b [95% CI]**	**P**
MGM's education <O-Level (c686a)	2721	1815 [1026, 2604]	**6.8×10^{-6}**	2277	1929 [1083, 2775]	**8.2×10^{-6}**
MGF's year of birth (c783yr)	3076	119.6 [77.1, 162.0]	**3.6×10^{-8}**	2277	107.5 [59.7, 155.2]	**1.1×10^{-5}**
Mother's year of birth (mzyear)	3573	225.1 [148.6, 301.6]	**8.7×10^{-9}**			
Mother was born in Avon (d245)	3177	1730 [998.8, 2462]	**3.7×10^{-6}**			
Mother born post-term (dw034)	3362	2700 [904.4, 4496]	**0.003**	2277	2793 [720.8, 4865]	**0.008**
Mother was breast fed (c624)	3009	-1446 [-2229, -662.4]	**3.0×10^{-4}**			
Mother was badly scalded aged <6 (d085_0)	3380	4029 [826.7, 7232]	**0.014**	2277	4556 [684.8, 8427]	**0.021**
Mother nearly drowned aged <6 (d097_0)	3391	4363 [467.7, 8258]	**0.028**	2277	5250 [523.3, 9977]	**0.030**
						R^2=2.52%

Supplementary Analysis AM7. Mother in childhood excluding early measures (tables AM3, AM4 and AM5 combined)

		**Unadjusted**			**Adjusted**	
**Variable**	**N**	**b [95% CI]**	**P**	**N**	**b [95% CI]**	**P**
MGM in household when aged 6-11 (d421)	3449	-2563 [-4257, -868.8]	**0.003**	2792	-2179 [-4129, -229.1]	**0.029**
Stepfather in household when aged 6-11 (d436)	3449	2329 [87.0, 4571]	**0.042**			
Grandfather in household when aged 6-11 (d454)	3449	2588 [556.2, 4619]	**0.013**	2792	3037 [760.2, 5313]	**0.009**
Mother wetting during day mid-childhood (d274b)	3449	4455 [627.7, 8283]	**0.023**			
Mother fractured arm when aged 6-11 (d091_6)	3309	2685 [1015, 4355]	**0.002**	2792	2215 [431.7, 3997]	**0.015**
Mother started having periods aged 6-11 (d010_6)	3121	2227 [1261, 3193]	**6.4×10^{-6}**	2792	2092 [1069, 3115]	**6.2×10^{-5}**
Mother began smoking when aged 12-15 (b651_12)	3483	2518 [1373, 3663]	**1.7×10^{-5}**	2792	2273 [987.8, 3559]	**0.001**
Mother's parent had accident aged 12-15 (c410a)	3462	2175 [385.4, 3964]	**0.017**	2792	2184 [206.0, 4163]	**0.030**
Mother attended <3 schools (c440)	3344	-894.0 [-1626, -162.5]	**0.017**	2792	-995.6 [-1795, -196.2]	**0.015**
Mother attended a special school (d273)	3401	4358 [1032, 7684]	**0.010**			
Mother lived with her grandparents (d392)	3429	3066 [1190, 4943]	**0.001**			
						R^2=2.11%

Supplementary Analysis AM8. The maternal grandparents and mother's childhood (tables AM6 and AM7 combined)

	Unadjusted			Adjusted		
**Variable**	**N**	**b [95% CI]**	**P**	**N**	**b [95% CI]**	**P**
MGM's education <O-Level (c686a)	2721	1815 [1026, 2604]	**6.8 x 10^{-6}**	2100	1984 [1106, 2863]	**9.9 x 10^{-6}**
MGF's year of birth (c783yr)	3076	119.6 [77.1, 162.0]	**3.6 x 10^{-8}**	2100	96.2 [46.2, 146.3]	**1.7 x 10^{-4}**
Mother born post-term (dw034)	3362	2700 [904.4, 4496]	**0.003**	2100	2270 [73.4, 4467]	**0.043**
Mother was badly scalded aged <6 (d085_0)	3380	4029 [826.7, 7232]	**0.014**	2100	5417 [1402, 9431]	**0.008**
Mother nearly drowned aged <6 (d097_0)	3391	4363 [467.7, 8258]	**0.028**			
MGM in household when aged 6-11 (d421)	3449	-2563 [-4257, -868.8]	**0.003**			
Grandfather in household when aged 6-11 (d454)	3449	2588 [556.2, 4619]	**0.013**	2100	4462 [1783, 7142]	**0.001**
Mother fractured arm when aged 6-11 (d091_6)	3309	2685 [1015, 4355]	**0.002**			
Mother started having periods aged 6-11 (d010_6)	3121	2227 [1261, 3193]	**6.4 x 10^{-6}**	2100	1736 [593.3, 2878]	**0.003**
Mother began smoking when aged 12-15 (b651_12)	3483	2518 [1373, 3663]	**1.7 x 10^{-5}**	2100	2110 [612.0, 3609]	**0.006**
Mother's parent had accident aged 12-15 (c410a)	3462	2175 [385.4, 3964]	**0.017**	2100	2787 [567.0, 5008]	**0.014**
Mother attended <3 schools (c440)	3344	-894.0 [-1626, -162.5]	**0.017**			
						R^2=3.96%

The Paternal Line

Supplementary Analysis AP1: The paternal grandparents prior to the birth of the study father

Variable	Unadjusted N	Unadjusted b [95% CI]	Unadjusted P	Adjusted N	Adjusted b [95% CI]	Adjusted P
PGM's year of birth (pb420yr)	2061	77.9 [23.4, 132.4]	**0.005**			
PGM's education <O-Level (pb359a)	2279	1324 [470.9, 2178]	**0.002**			
PGM's age at father's birth (pb420)	2536	-76.5 [-146.0, -7.1]	**0.031**			
PGF's year of birth (pb431yr)	1994	55.5 [6.3, 104.7]	**0.027**	1898	56.1 [5.2, 107.0]	**0.031**
PGF's education <O-Level (pb376a)	2289	874.6 [29.0, 1720]	**0.043**			
PGF ever smoked (pb433)	2632	1031 [65.6, 1997]	**0.036**			
PGF's age at father's birth (pb431)	2454	-44.7 [-104.4, 15.0]	0.142			
PGF's social class (pb_sc_pgf)	2664	797.3 [475.4, 1119]	**1.3×10^{-6}**	1898	770.3 [394.0, 1147]	**6.2×10^{-5}**
						R^2=1.16%

Supplementary Analysis AP1x: The paternal grandparents (significant factors from analysis AP1, with grandmother smoking in pregnancy and interaction with child's sex)

Variable	Unadjusted N	Unadjusted b [95% CI]	Unadjusted P	Adjusted N	Adjusted b [95% CI]	Adjusted P
PGF's year of birth (pb431yr)	1994	55.5 [6.3, 104.7]	**0.027**	1879	42.5 [-8.3, 93.2]	0.101
PGF's social class (pb_sc_pgf)	2664	797.3 [475.4, 1119]	**1.3×10^{-6}**	1879	819.6 [445.2, 1194]	**1.9×10^{-5}**
PGM smoked when pregnant with father (pb423)	2830	807.2 [19.5, 1595]	**0.045**	1879	-702.3 [-2207, 802.5]	0.360
Child's sex (kz021)	3843	4492 [3801, 5183]	**1.8×10^{-36}**	1879	3318 [2147, 4489]	**3.2×10^{-8}**
Interaction between PGM smoking and child's sex	2830	1977 [387.6, 3566]	**0.015**	1879	1673 [-256.0, 3603]	0.089
						R^2=4.86%

Supplementary Analysis AP1xx: The paternal grandparents (as table AP1x but with year of birth of PGF removed)

		Unadjusted			Adjusted	
**Variable**	**N**	**b [95% CI]**	**P**	**N**	**b [95% CI]**	**P**
PGF's social class (pb_sc_pgf)	2664	797.3 [475.4, 1119]	**1.3×10^{-6}**	2628	803.6 [483.5, 1124]	**9.0×10^{-7}**
PGM smoked when pregnant with father (pb423)	2830	807.2 [19.5, 1595]	**0.045**	2628	-949.0 [-2247, 349.0]	0.152
Child's sex (kz021)	3843	4492 [3801, 5183]	**1.8×10^{-36}**	2628	3346 [2326, 4367]	**1.5×10^{-10}**
Interaction between PGM smoking and child's sex	2830	1977 [387.6, 3566]	**0.015**	2628	2361 [719.6, 4002]	**0.005**
						R^2=5.25%

Supplementary Analysis AP2: Exposures of father from birth to age 11

		Unadjusted			Adjusted	
**Variable**	**N**	**b [95% CI]**	**P**	**N**	**b [95% CI]**	**P**
Father was born in Avon (pa255)	2439	1192 [347.8, 2036]	**0.006**	2005	1262 [343.0, 2182]	**0.007**
Father nearly drowned when aged 6-11 (pa127_6)	2600	3326 [116.0, 6537]	**0.042**	2005	3571 [97.4, 7045]	**0.044**
Father had head injury when aged 6-11 (pa151_6)	2291	1845 [436.9, 3252]	**0.010**	2005	2191 [723.3, 3659]	**0.003**
Father had a stepfather at home aged 6-11 (pa436)	2686	3415 [576.1, 6253]	**0.018**			
Father's father died when aged 6-11 (pa631_6)	2686	3604 [-547.1, 7755]	0.089			
Father started smoking when aged <11 (b691_11)	3484	4300 [338.8, 8262]	**0.033**	2005	11560 [5391, 17730]	**2.4×10^{-4}**
Father often absent from school aged <11 (pb493)	2851	1741 [51.6, 3431]	**0.043**			
Father absent from school: illness aged <11 (pb495)	2918	2211 [338.2, 4085]	**0.021**			
Father often truanted from school age <11 (pb497)	2918	4754 [1318, 8189]	**0.007**	2005	5611 [1019, 10203]	**0.017**
						R^2=1.95%

Supplementary Analysis AP3: Remaining childhood exposures of father

Variable	Unadjusted N	Unadjusted b [95% CI]	Unadjusted P	Adjusted N	Adjusted b [95% CI]	Adjusted P
Father's father present when aged 12-16 (pa425)	2686	-1670 [-2846, -494.3]	**0.005**			
Father had stepfather at home aged 12-16 (pa437)	2686	2716 [536.5, 4895]	**0.015**	2371	2294 [45.8, 4543]	**0.046**
Father started smoking when aged 12-15 (b691_12)	3484	1937 [541.3, 3333]	**0.007**			
Father often absent from school aged 11+ (pb494)	2849	2584 [1248, 3921]	**1.5×10^{-4}**	2371	2844 [1266, 4422]	**4.2×10^{-4}**
Father absent from school: illness aged 11+ (pb496)	2918	3956 [1306, 6606]	**0.003**			
Father often truanted from school age 11+ (pb498)	2918	2161 [703.7, 3619]	**0.004**			
Degree to which father disliked school (pb491)	2878	581.6 [207.6, 955.7]	**0.002**			
Father did not find school valuable (pb492)	2879	476.3 [87.3, 865.4]	**0.016**			
Father lived with foster parents (pa395)	2627	6215 [661.7, 11769]	**0.028**			
Father spent time in a children's home (pa402a)	2578	7023 [1999, 12046]	**0.006**	2371	8799 [2896, 14702]	**0.004**
Father's mother was always stable (pa750)	2586	1037 [215.2, 1858]	**0.013**	2371	1107 [257.1, 1957]	**0.011**
						R^2=1.40%

Supplementary Analysis AP4: Father's childhood (tables AP2 & AP3 combined)

Variable	Unadjusted N	Unadjusted b [95% CI]	Unadjusted P	Adjusted N	Adjusted b [95% CI]	Adjusted P
Father was born in Avon (pa255)	2439	1192 [347.8, 2036]	**0.006**	1902	1169 [232.9, 2105]	**0.014**
Father nearly drowned when aged 6-11 (pa127_6)	2600	3326 [116.0, 6537]	**0.042**	1902	4063 [521.8, 7604]	**0.025**
Father had head injury when aged 6-11 (pa151_6)	2291	1845 [436.9, 3252]	**0.010**	1902	2318 [834.6, 3802]	**0.002**
Father started smoking when aged <11 (b691_11)	3484	4300 [338.8, 8262]	**0.033**	1902	10166 [3719, 16614]	**0.002**
Father often truanted from school age <11 (pb497)	2918	4754 [1318, 8189]	**0.007**	1902	5457 [538.7, 10377]	**0.030**
Father had stepfather at home aged 12-16 (pa437)	2686	2716 [536.5, 4895]	**0.015**			
Father often absent from school aged 11+ (pb494)	2849	2584 [1248, 3921]	**1.5×10^{-4}**	1902	2681 [818.5, 4543]	**0.005**
Father spent time in a children's home (pa402a)	2578	7023 [1999, 12046]	**0.006**	1902	6961 [159.7, 13762]	**0.045**
Father's mother was always stable (pa750)	2586	1037 [215.2, 1858]	**0.013**	1902	1265 [317.6, 2212]	**0.009**
						R^2=3.27%

Supplementary Analysis AP5: Father's childhood and the paternal grandparents (tables AP1 and AP4 combined)

Variable	Unadjusted N	Unadjusted b [95% CI]	Unadjusted P	Adjusted N	Adjusted b [95% CI]	Adjusted P
Father was born in Avon (pa255)	2439	1192 [347.8, 2036]	**0.006**			
Father nearly drowned when aged 6-11 (pa127_6)	2600	3326 [116.0, 6537]	**0.042**			
Father had head injury when aged 6-11 (pa151_6)	2291	1845 [436.9, 3252]	**0.010**	1975	2061 [632.4, 3490]	**0.005**
Father started smoking when aged <11 (b691_11)	3484	4300 [338.8, 8262]	**0.033**	1975	11224 [5231, 17218]	**2.5×10^{-4}**
Father often truanted from school age <11 (pb497)	2918	4754 [1318, 8189]	**0.007**			
Father often absent from school aged 11+ (pb494)	2849	2584 [1248, 3921]	**1.5×10^{-4}**	1975	2803 [1083, 4523]	**0.001**
Father spent time in a children's home (pa402a)	2578	7023 [1999, 12046]	**0.006**			
Father's mother was always stable (pa750)	2586	1037 [215.2, 1858]	**0.013**			
PGF's social class (pb_sc_pgf)	2664	797.3 [475.4, 1119]	**1.3×10^{-6}**	1975	642.5 [278.8, 1006]	**0.001**
PGM smoked when pregnant with father (pb423)	2830	807.2 [19.5, 1595]	**0.045**	1975	-1340 [-2807, 127.0]	0.073
Child's sex (kz021)	3843	4492 [3801, 5183]	**1.8×10^{-36}**	1975	2553 [1399, 3708]	**1.5×10^{-5}**
Interaction between PGM smoking and child's sex	2830	1977 [387.6, 3566]	**0.015**	1975	3211 [1340, 5082]	**0.001**
						R^2=6.33%
