# Supplementary material for: Investigating Possible Trans/Intergenerational Associations With Obesity in Young Adults Using an Exposome Approach
Source: Front Genet. 2019 Apr 5;10:314. doi: 10.3389/fgene.2019.00314 (PMC6459952; doi:10.3389/fgene.2019.00314)
Supplement: Supplementary file 2 [file Data_Sheet_2.PDF]

Supplement B. Summary of results

The Maternal Line

Supplementary Table B1. The maternal grandparents (G0)

Variable considered	a	b	c	d	e
MGM's year of birth	****	-	-	****	**
MGM's education level	****	****	****	**	*
MGM's age at mother's birth	***	-	-	**	
MGM's social class	****	-	-	**	
MGF's year of birth	****	****	****	****	**
MGF's education level	****	-	-	**	**
MGF ever smoked	***	-	-	**	
MGF's age at mother's birth	****	-	-	***	
MGF's social class	****	-	-	***	*

Variables that are associated with mean fat mass in the following categories: (a) all unadjusted; (b) all adjusted in subgroup model; (c) adjusted in final model; (d) female grandchildren (G2) unadjusted; (e) male grandchildren (G2) unadjusted. *P<0.05; **P<0.01; ***P<0.001; ****P<0.0001.

.

Supplementary Table B2. The mother (G1) in early childhood

Variable considered	a	b	c	d	e
M's year of birth	****	****	****	****	***
M was born in Avon	****	**	-	**	**
M was born post-term	**	**	*		
M was breast fed	****	**	-	*	*
M was badly scalded aged 6-11	*	*	**		*
M had a head injury aged 6-11	*	-	-		
M nearly drowned aged 6-11	*	**	*		
M had a serious accident 6-11	*	-	-		*
M lived with a stepfather 6-11	-	-	-	*	
M's parents divorced/separated Aged 6-11	*	-			

Variables that are associated with mean fat mass in the following categories: (a) all unadjusted; (b) all adjusted in subgroup model; (c) adjusted in final model; (d) female grandchildren (G2) unadjusted; (e) male grandchildren (G2) unadjusted. *P<0.05; **P<0.01; ***P<0.001; ****P<0.0001.

Supplementary Table B3. The mother (G1) in mid-childhood

Variables considered	a	b	c	d	e
MGM in household when M 6-11	**	**	-	***	
MGF in household when M 6-11	****	-	-	****	
Step-father in household when M 6-11	*	*	-	*	
M's grandfather in household when M 6-11	*	**	**	**	
M had history of daytime wetting 6-11	*	**	-	*	
M fractured arm aged 6-11	**	**	-	*	
M's periods started before age 12	****	****	**	***	**

Variables that are associated with mean fat mass in the following categories: (a) all unadjusted; (b) all adjusted in subgroup model; (c) adjusted in final model; (d) female grandchildren (G2) unadjusted; (e) male grandchildren (G2) unadjusted. *P<0.05; **P<0.01; ***P<0.001; ****P<0.0001.

Supplementary Table B4. The mother (G1) in late-childhood

Variables considered	a	b	c	d	e
MGM in household when M 12-15	**	-	-	**	**
MGF in household when M 12-15	*	-	-		
M had a head injury when M 12-15	*	-	-		
M began smoking aged 12-15	****	****	**	***	***
M admitted to hospital aged 12-15	*	-	-		
M's parent had an accident when M 12-15	*	*	*		
M became pregnant aged 12-15	*	-	-		
M suspended from school aged 12-15	*	-	-		

Variables that are associated with mean fat mass in the following categories: (a) all unadjusted; (b) all adjusted in subgroup model; (c) adjusted in final model; (d) female grandchildren (G2) unadjusted; (e) male grandchildren (G2) unadjusted. *P<0.05; **P<0.01; ***P<0.001; ****P<0.0001.

Supplementary Table B5. The mother (G1) in other aspects of childhood (<17)

Variables considered	a	b	c	d	e
M attended <3 schools	*	*	-		*
M attended a special school	*	*	-	**	
M was in care	**	-	-	**	
M lived with her grandparents	**	**	-	*	*
M stayed in a children's home	*	-	-	*	
M's mother was over-protective	*	-	-		

Variables that are associated with mean fat mass in the following categories: (a) all unadjusted; (b) all adjusted in subgroup model; (c) adjusted in final model; (d) female grandchildren (G2) unadjusted; (e) male grandchildren (G2) unadjusted. *P<0.05; **P<0.01; ***P<0.001; ****P<0.0001.

The Paternal Line

Supplementary Table B6. The paternal grandparents (G0)

Variable considered	a	b	c	d	e
PGM's year of birth	**	-	-	**	
PGM's education level	**	-	-	***	
PGM's age at father's birth	*	-	-	**	
PGM's social class	-	-	-	-	
PGF's year of birth	*	****	-		
PGF's education level	*	-	-	**	
PGF ever smoked	*	-	-		*
PGF's age at father's birth	-	-	-		
PGF's social class	****	****	***	****	*
PGM smoked in pregnancy X sex of G2	*	(*)	***	DNA	DNA

Variables that are associated with mean fat mass in the following categories: (a) all unadjusted; (b) all adjusted in subgroup model; (c) adjusted in final model; (d) female grandchildren (G2) unadjusted; (e) male grandchildren (G2) unadjusted. DNA = Does not apply. *P<0.05; **P<0.01; ***P<0.001; ****P<0.0001.

Supplementary Table B7. The father (G1) in early/mid childhood

Variables considered	a	b	c	d	e
F born in Avon	**	**	-		
F nearly drowned when 6-11	*	*	-		
F had head injury aged 6-11	*	**	**		
F had step-father at home 6-11	*		-	**	
F's father died when he was 6-11	(*)		-	*	
F started smoking regularly <11	*	****	****		*
F often absent from school <11	*		-		
F often absent from school due to illness <11	*		-		**
F often truanted from school aged <11	**	*	-	*	

Variables that are associated with mean fat mass in the following categories: (a) all unadjusted; (b) all adjusted in subgroup model; (c) adjusted in final model; (d) female grandchildren (G2) unadjusted; (e) male grandchildren (G2) unadjusted. (*)P<0.10; *P<0.05; **P<0.01; ***P<0.001; ****P<0.0001.

Supplementary Table B8. The father (G1) in other childhood variables

**Variables considered**	**a**	**b**	**c**	**d**	**e**
PGF in household when F aged 12-15	**	-	-	****	
F’s stepfather in household when F 12-15	*	*	-	*	
F started smoking when aged 12-15	**	-	-	*	
F often absent from school when aged 11+	****	****	***	**	*
F absent from school because of illness 11+	**	-	-		***
F truanted from school aged 11+	**	-	-	*	
Degree to which F disliked school	**	-	-	***	
Degree to which F did not find school valuable	*	-	-	**	
F lived with foster parents < 17	*	-	-		*
F spent time in a children’s home <17	**	**	-		*
F’s mother’s degree of instability	*	*	-		*

Variables that are associated with mean fat mass in the following categories: (a) all unadjusted; (b) all adjusted in subgroup model; (c) adjusted in final model; (d) female grandchildren (G2) unadjusted; (e) male grandchildren (G2) unadjusted. *P<0.05; **P<0.01; ***P<0.001; ****P<0.0001.
