# Supplementary material for: Investigating Possible Trans/Intergenerational Associations With Obesity in Young Adults Using an Exposome Approach
Source: Front Genet. 2019 Apr 5;10:314. doi: 10.3389/fgene.2019.00314 (PMC6459952; doi:10.3389/fgene.2019.00314)
Supplement: Supplementary file 3 [file Data_Sheet_3.PDF]

Supplement C. The data used in the analyses

The Maternal line

M1. Characteristics of the Maternal Grandparents (G0) at the time of birth of the study mother

MGM's year of birth – treated as continuous

MGM's education level (taking the current equivalent qualifications) - ≤ O-level; > O-level

MGM's age at the birth of the study mother – treated as continuous

MGM's social class – based on her occupation and categorised as I, II, IIINM; IIIM; IV and V, and treated as continuous. [N.B. there were categorisations into occupation type and social group, but both were left out of these analyses as they were also based on a classification of the occupation and gave essentially the same results as social class].

MGM's ethnic origin – divided as white and non-white.

MGM's primiparity at the birth of the mother – yes; no - estimated from whether the mother had any older siblings.

MGM was a smoker – yes; no

MGM smoked when pregnant with the study mother – yes[a]; no

MGF's year of birth – treated as continuous

MGF's education level (taking the current equivalent qualifications) - ≤ O-level; > O-level

MGF's age at the birth of the study mother – treated as continuous

MGF's social class – based on his occupation and categorised as I, II, IIINM; IIIM; IV and V, and treated as continuous. [N.B. there were categorisations into occupation type and social group, but both were left out as they were also based on a classification of the occupation, and gave essentially the same results as social class].

MGF's ethnic origin – divided as white and non-white.

MGF was a smoker – yes; no

{14 variables}

M2. Characteristics of the Mother at Birth (G1)

Birthweight – this was frequently missing. It has therefore been omitted from these analyses.

Gestation – this variable identified whether the mother had been born 'more than 3 weeks early' or '3 weeks late'. This identifies the mothers born preterm and those born post-term, and thus comprises two variables.

Structural defect present at birth

Ethnic origin – white; non-white

Birthmark present – yes; no

M's year of birth – continuous

Place of residence at birth – characterised as Avon; not Avon

Whether adopted soon after birth – yes; no

Whether breast fed

No. older brothers – 0, 1, 2, 3+ treated as continuous

No. older sisters – 0, 1, 2, 3+ treated as continuous

No. older siblings – 0, 1, 2, 3, 4+ treated as continuous

{12 variables}

M3. Characteristics of her early childhood (< 6 years of age)

Accidents and injuries: there were specific yes/no questions about the following:

- Badly burnt; badly scalded; ingested medicines; fractured arm/hand; fractured leg/foot; nearly drowned; road traffic accident; accident on bicycle; head injury; badly cut; bad fall. A derived variable was used to identify whether there were positive replies to any of these questions. {12 questions}

Composition of M's household during these early years elicited whether any of the following were present (yes/no):

- her mother; her father; a brother; a sister; step-father; step-brother; step-sister; M's mother's partner; M's father's partner; M's grandmother; a family friend. {11 questions}

Traumatic events that occurred in this time period (yes/no):

- her parents divorced/separated; she was sexually abused; her mother died; her father died;
- her recollections of happiness/unhappiness during the period – 4-point-scale from very happy to quite or very unhappy – treated as continuous.
  {5 questions}

M4. Characteristics of her mid-childhood (6-11 years of age)

Accidents and injuries: there were specific yes/no questions about the following:

- Badly burnt; badly scalded; ingested medicines; fractured arm/hand; fractured leg/foot; nearly drowned; road traffic accident; accident on bicycle; head injury; badly cut; bad fall. A derived variable was used to identify whether there were positive replies to any of these questions. {12 questions}

Composition of M's household during these early years elicited whether any of the following were present (yes/no):

- her mother; her father; a brother; a sister; step-father; step-brother; step-sister; M’s mother’s partner; M’s father’s partner; M’s grandmother; M’s grandfather; a family friend. {12 questions}

Traumatic events that occurred in this time period (yes/no):

- her parents divorced/separated; she was adopted; she was sexually abused; her mother died; her father died;
- her recollections of happiness/unhappiness during the period – 5-point-scale from very happy to very unhappy – treated as continuous. {6 questions}

Other features occurring in this time period:

- her periods started; she was wetting the bed; she was wetting during the day; she started smoking. {4 questions}

M5. Characteristics of her late childhood (12-15 years of age)

Accidents and injuries: there were specific yes/no questions about the following:

- badly burnt; badly scalded; ingested medicines; fractured arm/hand; fractured leg/foot; nearly drowned; road traffic accident; accident on bicycle; head injury; badly cut; bad fall. A derived variable was used to identify whether there were positive replies to any of these questions. {12 questions}

Composition of M’s household during these early years elicited whether any of the following were present (yes/no):

- her mother; her father; a brother; a sister; step-father; step-brother; step-sister; M’s mother’s partner; M’s father’s partner; M’s grandmother; M’s grandfather; a family friend. {12 questions}

Traumatic events that occurred in this time period (yes/no):

- her parents divorced/separated; she was adopted; she was sexually abused; her mother died; her father died;
- her recollections of happiness/unhappiness during the period – 5-point-scale from very happy to very unhappy – treated as continuous. {6 questions}

Other features occurring in this time period:

- she started smoking. {1 question}

M6. Other characteristics of her childhood (< 17 years of age)

- life events occurring during childhood – a number of questions were asked with an instruction to weight the degree to which they were affected by the event, ranging from ‘not at all’ to ‘a lot’ (see the Addendum for the description of the details and the psychometrics). Here we have just considered whether the event occurred in a yes/no format. {31 questions}

- schooling and treatments – questions on the number of schools attended - <3, 3, 4+ ; whether attended a special school; was at a boarding school; was treated by a physiotherapist; seen by a child psychiatrist; or treated by a speech therapist. {6 questions}
- where and with whom she lived – options in a yes/no format were: with grandparents; with other relatives; with friends; with foster parents; in a Children's Home; whether she left home before age 18. She was also asked how many different persons brought her up, and a derived variable identified <2, 2, 3, 4+ persons. {7 items}
- relationship with her mother – this was measured using two scores from the Parent Bonding Index (see Addendum for details). She was also asked to rate the stability of her parents using options: always/mostly/ rarely or never for her mother and for her father. A derived variable was used to assess the stability of the home into 4 categories: very stable; fairly stable; unstable; very unstable. {5 items}
- other details include: No. younger brothers – 0, 1, 2, 3+ treated as continuous; No. younger sisters – 0, 1, 2, 3+ treated as continuous; No. younger siblings – 0, 1, 2, 3, 4+ treated as continuous; Total number of older and younger siblings – 0, 1, 2, 3+ treated as continuous. {4 items}

The Paternal line

P1. Characteristics of the Paternal Grandparents (G0) at the time of birth of the study Father

PGM's year of birth – treated as continuous

PGM's education level (taking the current equivalent qualifications) - $\leq$ O-level; > O-level

PGM's age at the birth of the study mother – treated as continuous

PGM's social class – based on her occupation and categorised as I, II, IIINM; IIIM; IV and V, and treated as continuous. [N.B. there were categorisations into occupation type and social group, but both were left out of these analyses as they were also based on a classification of the occupation and gave essentially the same results as social class].

PGM's ethnic origin – divided as white and non-white.

PGM's primiparity at the birth of the mother – yes; no - estimated from whether the mother had any older siblings.

PGM was a smoker – yes; no

PGM smoked when pregnant with the study mother – yes[a]; no

PGF's year of birth – treated as continuous

PGF's education level (taking the current equivalent qualifications) - $\leq$ O-level; > O-level

PGF's age at the birth of the study mother – treated as continuous

PGF's social class – based on his occupation and categorised as I, II, IIINM; IIIM; IV and V, and treated as continuous. [N.B. there were categorisations into occupation type and social

group, but both were left out as they were also based on a classification of the occupation, and gave essentially the same results as social class].

PGF's ethnic origin – divided as white and non-white.

PGF was a smoker – yes; no. {14 variables}

P2. Characteristics of the Father at Birth (G1)

Birthweight – this was frequently missing. It has therefore been omitted from these analyses.

Gestation – this variable identified whether the father had been born 'more than 3 weeks early' or '3 weeks late'. This identifies the fathers born preterm and those born post-term, and thus comprises two variables.

Structural defect present at birth

Ethnic origin – white; non-white

Birthmark present – yes; no

Place of residence at birth – characterised as Avon; not Avon

Whether adopted soon after birth – yes; no

Whether breast fed

No. older brothers – 0, 1, 2, 3+ treated as continuous

No. older sisters – 0, 1, 2, 3+ treated as continuous

No. older siblings – 0, 1, 2, 3, 4+ treated as continuous. {11 variables}

P3. Characteristics of his early childhood (< 6 years of age)

Accidents and injuries: there were specific yes/no questions about the following:

- badly burnt; badly scalded; ingested medicines; fractured arm/hand; fractured leg/foot; nearly drowned; road traffic accident; accident on bicycle; head injury; badly cut; bad fall. A derived variable was used to identify whether there were positive replies to any of these questions. {12 questions}

Composition of F's household during these early years elicited whether any of the following were present (yes/no):

- his mother; his father; a brother; a sister; step-father; step-brother; step-sister; F's mother's partner; F's father's partner; F's grandmother; F's grandfather; a family friend. {12 questions}

Traumatic events that occurred in this time period (yes/no):

- his parents divorced/separated; he was adopted; he was neglected; he was sexually abused; his mother died; his father died;

- his recollections of happiness/unhappiness during the period – 4-point-scale from very happy to quite or very unhappy – treated as continuous. {7 questions}

P4. Characteristics of his mid-childhood (6-11 years of age)

Accidents and injuries: there were specific yes/no questions about the following:

- badly burnt; badly scalded; ingested medicines; fractured arm/hand; fractured leg/foot; nearly drowned; road traffic accident; accident on bicycle; head injury; badly cut; bad fall. A derived variable was used to identify whether there were positive replies to any of these questions. {12 questions}

Composition of F’s household during these early years elicited whether any of the following were present (yes/no):

- his mother; his father; a brother; a sister; step-father; step-brother; step-sister; F’s mother’s partner; F’s father’s partner; F’s grandmother; F’s grandfather; a family friend. {12 questions}

Traumatic events that occurred in this time period (yes/no):

- his parents divorced/separated; he was adopted; he was neglected; he was sexually abused; his mother died; his father died;
- his recollections of happiness/unhappiness during the period – 5-point-scale from very happy to very unhappy – treated as continuous. {7 questions}

Other features occurring in this time period:

- he started smoking; he was frequently absent from school; he was frequently absent from school because of illness; he frequently truanted from school. {4 questions}
-

P5. Characteristics of his late childhood (12-15 years of age)

Accidents and injuries: there were specific yes/no questions about the following:

- badly burnt; badly scalded; ingested medicines; fractured arm/hand; fractured leg/foot; nearly drowned; road traffic accident; accident on bicycle; head injury; badly cut; bad fall. A derived variable was used to identify whether there were positive replies to any of these questions. {12 questions}

Composition of F’s household during these early years elicited whether any of the following were present (yes/no):

- his mother; his father; a brother; a sister; step-father; step-brother; step-sister; F’s mother’s partner; F’s father’s partner; F’s grandmother; F’s grandfather; a family friend. {12 questions}

Traumatic events that occurred in this time period (yes/no):

- his parents divorced/separated; he was adopted; he was neglected; he was sexually abused; his mother died; his father died;

- his recollections of happiness/unhappiness during the period – 5-point-scale from very happy to very unhappy – treated as continuous. {7 questions}

Other features occurring in this time period:

- he started smoking. {1 question}

P6. Other characteristics of his childhood (< 17 years of age)

- life events occurring during childhood – a number of questions were asked with an instruction to weight the degree to which they were affected by the event, ranging from 'not at all' to 'a lot' (see the Addendum for the description of the details and the psychometrics). Here we have just considered whether the event occurred in a yes/no format. {30 questions}
- schooling and treatments – questions on the number of schools attended - <3, 3, 4+ ; whether attended a special school; was at a boarding school; was frequently absent from school aged 11+; was frequently absent because of illness 11+; frequently truanted from school aged 11+; liked school – 5 point scale; found school a valuable experience – 5 point scale; was treated by a physiotherapist; seen by a child psychiatrist; or treated by a speech therapist. {11 questions}
- where and with whom he lived – options in a yes/no format were: with grandparents; with other relatives; with friends; with foster parents; in care; in custody; in a Children's Home; whether he left home before age 18. He was also asked how many different persons brought him up, and a derived variable identified <2, 2, 3, 4+ persons. {9 items}
- relationship with his mother – this was measured using two scores from the Parent Bonding Index (see Addendum for details). He was also asked to rate the stability of his parents using options: always/mostly/ rarely or never for his mother and for his father. A derived variable was used to assess the stability of the home into four categories: very stable; fairly stable; unstable; very unstable. {5 items}
- other details include: No. younger brothers – 0, 1, 2, 3+ treated as continuous; No. younger sisters – 0, 1, 2, 3+ treated as continuous; No. younger siblings – 0, 1, 2, 3, 4+ treated as continuous; Total number of older and younger siblings – 0, 1, 2, 3+ treated as continuous. {4 items}

Addendum. The psychological scales used

Relationship with her mother

The relationship of the mother with her own mother (or mother figure) was elicited using a 22-item set of questions modified from the original Parent-Bonding Instrument (PBI). Respondents reported the quality of their relationship with their mother in childhood on the care and over protectiveness sub-scales.(Parker, Tupling, & Brown, 1979). Previous research supports the validity and reliability of this scale, particularly its association with depression and the validity of retrospective reports (Parker, 1981).

The original Parental Bonding Instrument (Parker, Tupling and Brown 1979) had been adapted by Gamsa (1987) to reword the statements that had produced double negatives in the original.

During the course of ALSPAC's piloting it became obvious that our mothers were unhappy with the original options for responses (very like, moderately like, moderately unlike, very unlike) and they have been changed to: 'never', 'sometimes', 'usually'. In addition, three questions were omitted since they were almost identical to other questions in the scale and caused considerable annoyance to participants. The introduction to the 22 statements read as follows: 'we would like to know how you and your mother got on when you were a child. This will probably have varied over your childhood and in different situations, but we would like a general impression. Please tick the box to indicate how you mostly remember your mother in the first 16 years'.

Two scores were derived from these 22 questions: a 'maternal care' score, and an 'overprotective' score. Internal consistencies in this sample were .73 and .70 for care and over protectiveness, respectively (O'Connor et al 1999).

This instrument has been used (a) to show that the link between divorce of one's parents during childhood and adult depression and /or divorce was partly mediated through the quality of parent-child relations (O'Connor et al, 1999).

Gamsa, A. (1987) A note on a modification of the Parent Bonding Instrument. Br J Med Psychol, 60:291-294.

O`Connor TG, Thorpe K, Dunn J, Golding J, 'Parental divorce and adjustment in adulthood: findings from a community sample. The ALSPAC Study Team. Avon Longitudinal Study of Pregnancy and Childhood.', J Child Psychol Psychiatry 1999; 40 (5): 777-89

Parker G. Parental reports of depressives: An investigation of several explanations. J Affective Disorders 1981; 3: 131-140.

Parker, G., Tupling, H., and Brown, L.B. (1979) A Parental Bonding Instrument. British Journal of Medical Psychology, 1979, 52, 1-10.

Childhood life events

This comprised a set of questions of 31 specific items administered to the pregnant women in the third trimester of pregnancy, and 30 to their partners in mid-pregnancy [see PB

files]. Childhood was specified as being <17 years. The items were devised by the ALSPAC Study Team based on the earlier work of Coddington (1972). The items included four on deaths to parent(s), relative(s), sibling(s) and friend(s); three on serious illness to the participant as well as to a parent and sibling; three on experiencing a serious accident (to parent, participant, sibling); three on hospitalisation (to parent, participant and sibling); three concerning abuse to the participant (physical, sexual and emotional); seven relating to parents (separated, divorced, had serious arguments, remarried' imprisoned, mentally ill, family became poorer); and seven to the participant themselves (discovered that she/he was adopted, failed an important exam, moved to a new district, in trouble with the police, expelled or suspended from school, became physically deformed, became pregnant [or girlfriend became pregnant]). In addition, the mothers were asked if they had acquired a step-sibling, but this was accidentally omitted from the father's questionnaire. For both parents a question concerning anything else that occurred was asked, with a description written as text; these responses are not included. For each specified item there were five possible answers: '*yes, affected me a lot'*; '*yes, moderately affected'*; '*yes, mildly affected'*; '*yes, but did not affect me'*; '*no, did not happen'*.

From the answers to these questions two different scores were derived: (i) the total number of life events, and (ii) the weighted life events derived by scoring the five possible responses listed above as 4, 3, 2, 1, 0 respectively.

The perceived effects of each type of life event were calculated by summing the scores for that item and dividing by the number who had experienced that life event. This has been used to compare the scores given for different items between the mothers and their partners by Golding et al (2012). This showed a remarkable concordance between the two.

Validation

To support the validity of these ratings of abuse, Collishaw et al (2007) assessed associations with other relevant measures of parenting reported by mothers. Mean scores on the maternal care subscale of the Parental Bonding Instrument (Parker et al,1979), administered separately to mothers at an earlier assessment, were between 1.2 and 1.6 *SD* lower for those reporting emotional or physical cruelty than for the population as a whole. There were strong associations between reports of emotional and physical cruelty and reported sexual abuse. Finally, 95% of those reporting either moderate or severe emotional or physical cruelty also reported another form of abuse, or reported abuse at multiple time points, or indicated some other difficulty in their relationships with their parents (Parent Bonding Instrument care score ≤ -1 *SD*, violence between parents, or marked unpredictability of a parent.'

Coddington RD. The significance of life events as etiological factors in the diseases of children. J Psychosomatic Research 1972; 16: 7-18

Collishaw S, Dunn J, O`Connor TG, Golding J. Maternal childhood abuse and offspring adjustment over time. Dev Psychopathol 2007; 19 (2): 367-83

Parker G, Tupling H, Brown LB. A parent bonding instrument. British Journal of Medical Psychology 1979; 52: 1-10.

P1. Characteristics of the Maternal Grandparents (G0) at the time of birth of the study mother

PGM's year of birth – treated as continuous

PGM's education level (taking the current equivalent qualifications) - ≤ O-level; > O-level

PGM's age at the birth of the study mother – treated as continuous

PGM's social class – based on her occupation and categorised as I, II, IIINM; IIIM; IV and V, and treated as continuous. [N.B. there were categorisations into occupation type and social group, but both were left out of these analyses as they were also based on a classification of the occupation and gave essentially the same results as social class].

PGM's ethnic origin – divided as white and non-white.

PGM's primiparity at the birth of the mother – yes; no - estimated from whether the mother had any older siblings.

PGM was a smoker – yes; no

PGM smoked when pregnant with the study mother – yes[a]; no

PGF's year of birth – treated as continuous

PGF's education level (taking the current equivalent qualifications) - ≤ O-level; > O-level

PGF's age at the birth of the study mother – treated as continuous

PGF's social class – based on his occupation and categorised as I, II, IIINM; IIIM; IV and V, and treated as continuous. [N.B. there were categorisations into occupation type and social group, but both were left out as they were also based on a classification of the occupation, and gave essentially the same results as social class].

PGF's ethnic origin – divided as white and non-white.

PGF was a smoker – yes; no {14 variables}
